# Impact of body mass index on the efficacy of immune combination therapy in metastatic renal cell carcinoma: a multicenter study in Japan

**DOI:** 10.1007/s10147-025-02823-0

**Published:** 2025-08-20

**Authors:** Toshiki Anami, Takanobu Motoshima, Yuto Matsushita, Takahiro Kojima, Shimpei Yamashita, Hisanori Taniguchi, Keisuke Monji, Ryo Ishiyama, Yoshihide Kawasaki, Takuma Kato, Shuichi Tatarano, Kimihiko Masui, Eijiro Nakamura, Tomoyuki Kaneko, Makito Miyake, Hiroshi Kitamura, Hideaki Miyake, Tomomi Kamba, the Japan Urological Oncology Group

**Affiliations:** 1 Department of Urology, Faculty of Life Sciences, Graduate School of Medical Sciences, Kumamoto University, Honjo 1-1-1, Chuouku, Kumamoto 860-8556 Japan; 2 Department of Urology, Hamamatsu University School of Medicine, Shizuoka, Japan; 3 Department of Urology, Aichi Cancer Center, Aichi, Japan; 4 Department of Urology, Wakayama Medical University, Wakayama, Wakayama Japan; 5 Department of Urology and Andrology, Kansai Medical University, Osaka, Japan; 6 Department of Urology, Graduate School of Medical Sciences, Kyushu University, Fukuoka, Japan; 7 Department of Urology, Tokyo Women’s Medical University, Tokyo, Japan; 8 Department of Urology, Tohoku University Graduate School of Medicine, Miyagi, Japan; 9 Department of Urology, Faculty of Medicine, Kagawa University, Kagawa, Japan; 10 Department of Urology, Graduate School of Medical and Dental Sciences, Kagoshima University, Kagoshima, Japan; 11 Department of Urology, Kyoto University Graduate School of Medicine, Kyoto, Japan; 12 Department of Urology, National Cancer Center Hospital, Tokyo, Japan; 13 Department of Urology, Teikyo University School of Medicine, Tokyo, Japan; 14 Department of Urology, Nara Medical University, Nara, Japan; 15 Department of Urology, Faculty of Medicine, University of Toyama, Toyama, Japan; 16 Department of Urology, Kobe University Graduate School of Medicine, Hyogo, Japan

**Keywords:** Renal cell carcinoma (RCC), Immune combination therapy, Body mass index (BMI), Obesity paradox

## Abstract

**Background:**

Renal cell carcinoma (RCC) is a major urologic malignancy worldwide, with obesity recognized as a known risk factor. Interestingly, a higher body mass index (BMI) has been associated with improved outcomes in immunotherapy, a phenomenon termed the “obesity paradox.” This study investigates the influence of BMI on the effectiveness of immune combination therapies in Japanese patients with metastatic RCC.

**Methods:**

A retrospective study was conducted on 243 Japanese patients with metastatic RCC who received immune combination therapies between 2018 and 2022. Patients were stratified into two groups: non-overweight/obesity (BMI < 25 kg/m^2^) and overweight/obesity (BMI ≥ 25 kg/m^2^). Survival outcomes, including progression-free survival (PFS) and overall survival (OS), were compared between the groups.

**Results:**

There was no significant difference in PFS between the groups. However, the overweight/obesity group showed a trend toward longer OS, particularly in patients receiving IO–IO regimens (P = 0.011). In contrast, although no statistically significant difference was observed in the IO–TKI regimen, there was a trend toward prolonged OS in the non-overweight/obesity group. No significant differences in immune-related adverse events were observed between the groups.

**Conclusion:**

Higher BMI may be associated with better outcomes in immune combination therapy, especially with IO–IO regimens. These findings suggest that BMI could be a useful factor in optimizing RCC treatment. Further research with larger cohorts is needed to confirm these results and understand the mechanisms behind the “obesity paradox.”

**Supplementary Information:**

The online version contains supplementary material available at 10.1007/s10147-025-02823-0.

## Introduction

Renal cell carcinoma (RCC) accounts for ~ 2.2% of all malignant tumors in adults, with > 400,000 new cases diagnosed worldwide in 2022 that resulted in ~ 155,000 deaths [1]. The incidence of RCC is high in Western Europe, Northern Europe, and North America, but relatively low in Asia [2]. However, the incidence of RCC among Japanese Americans is higher than that among Japanese people living in Japan, suggesting that environmental factors such as lifestyle habits may be involved in the onset of RCC [3]. Obesity has been identified as a significant risk factor for renal cancer [4].

The treatment of metastatic RCC has undergone a major transformation over recent years, with the advent of immune checkpoint inhibitors (IOs). Nivolumab + ipilimumab, axitinib + pembrolizumab, axitinib + avelumab, cabozantinib + nivolumab, and lenvatinib + pembrolizumab have all shown promising results compared to sunitinib monotherapy and are now used as the first line for metastatic RCC [5–9].

Body mass index (BMI) is a commonly used indicator for evaluating whether a person’s weight is healthy for their height. Previous reports have suggested a complex relationship between BMI and immunotherapy outcomes [10]. Several studies have reported on the “obesity paradox,” wherein survival outcomes appear to be better in patients with higher BMIs [11–13], but the fundamental mechanism underlying this phenomenon has not yet been elucidated.

This study aimed to clarify the relationship between immune combination therapy and BMI in patients with metastatic RCC living in Japan. Our results might contribute to a better understanding of the factors that affect the efficacy of immunotherapy in patients with RCC and prove useful in the context of optimizing treatment strategies for this deadly malignancy.

## Patients and methods

### Participants

This retrospective multicenter study analyzed 243 patients who were clinically diagnosed with metastatic RCC between August 1, 2018 and January 31, 2022, at 34 facilities belonging to the Japan Urological Oncology Group, received immune combination therapy as their primary treatments, and received molecular targeted drugs as their secondary ones. In the dataset we selected, the immune combination therapy used was nivolumab + ipilimumab, axitinib + pembrolizumab, and axitinib + avelumab. The study was approved by the Institutional Review Board of Hamamatsu University School of Medicine (approval no.: 22-008), as well as the ethics committees of all 34 participating institutions. The requirement for informed consent was waived in favor of an opt-out approach.

### Evaluation

Patient data were retrospectively collected from each facility.

BMI was defined as weight (kg) divided by height (m) squared. Based on the classification published by the World Health Organization, a BMI of 25 kg/m^2^ or more is overweight or obese, so our statistical analysis was performed by dividing the patients into two groups: one with BMI values of < 25 kg/m^2^, and the other with values of ≥ 25 kg/m^2^. The former group was referred to as the non-overweight/obesity group (NOG), and the latter the overweight/obesity group (OG).

The best response was evaluated using the RECIST v1.1 criteria, and defined as either complete response (CR), partial response (PR), stable disease (SD), or progressive disease (PD).

Overall survival (OS) was defined as the period from the start of primary treatment until death, whereas progression-free survival (PFS) was defined as the period from the start of immune combination therapy until disease progression or death from any cause.

### Statistical analysis

Our statistical analysis was performed using GraphPad Prism 10 (GraphPad Software, Boston, MA, USA), with statistical significance set at P < 0.05. Comparisons between the two groups were performed using Mann–Whitney U and Fisher’s exact tests. PFS and OS were calculated using the Kaplan–Meier method, and differences between the two groups were compared using the log-rank test.

## Results

### Patient characteristics

The clinicopathological characteristics of the 243 study participants are presented in Table 1. The NOG group had 178 participants, with 65 in the OG group. The male/female ratios were 73.0/27.0% in the NOG group and 81.5/18.5% in the OG group (P = 0.18). The median ages were 66 and 67 years in the NOG and OG groups, respectively (P = 0.93). A tendency toward a higher proportion of multiple metastases was observed in the NOG group; however, this difference was not statistically significant. In the NOG group, a significantly higher proportion of patients had lung metastases (66.3% vs. 50.8%, NOG vs. OG; P = 0.036). No significant differences were observed in any other patient parameters between the two groups. IO–IO and IO–TKI were chosen as the primary treatments in 76.4% and 23.6%, respectively, of the patients in the NOG group, and in 80.0% and 20.0%, respectively of those in the OG one (P = 0.61). The median duration of first-line therapy was 6 months in the NOG group and 7 months in the OG group, with no statistically significant difference. The primarily reasons for discontinuing first-line therapy were disease progression (73.6% in the NOG group and 63.1% in the OG group) and adverse events (21.3% in the NOG group and 32.3% in the OG group), with no significant difference between groups. Most patients in both groups received axitinib or cabozantinib as second-line therapy.

**Table 1:** Patients’ characteristics

Patients n = 243	BMI < 25	BMI ≥ 25	p
Number	178	65	
Gender			0.18
Male	130 (73.0)	53 (81.5)	
Female	48 (27.0)	12 (18.5)	
Age, years			0.93
Median	66	67	
Range	30–84	24–86	
Histology			0.15
Clear cell	129 (72.5)	53 (81.5)	
Non-clear	41 (23.0)	12 (18.5)	
Unknown	8 (4.5)	0 (0)	
Past nephrectomy			0.88
Yes	104 (58.4)	39 (60.0)	
Number of metastatic sites			0.11
1	88 (49.4)	40 (61.5)	
≥ 2	90 (50.6)	25 (38.5)	
Sites of metastasis and recurrence			
Lung	118 (66.3)	33 (50.8)	0.036
Lymph node	49 (27.5)	19 (29.2)	0.87
Bone	52 (29.2)	16 (24.6)	0.52
Liver	22 (12.4)	9 (13.8)	0.83
Brain	6 (3.4)	4 (6.2)	0.46
Others	61 (34.3)	14 (21.5)	
IMDC risk stratification			0.62
Favorable risk	21 (11.8)	10 (15.4)	
Intermediate risk	102 (57.3)	39 (60.0)	
Poor risk	51 (28.7)	16 (24.6)	
Unknown	4 (2.2)	0 (0)	
First line therapy			0.61
IO–IO (nivolumab + ipilimumab)	137 (77.0)	52 (80.0)	
IO–TKI	41 (23.0)	13(20.0)	
axitinib + pembrolizumab	31	11	
axitinib + avelumab	10	2	
Duration of first line therapy, months			0.3
Median	6	7	
Range	0–48	0–39	
Reason for discontinuation first line therapy			0.22
Disease progression	131 (73.6)	41 (63.1)	
Adverse event	38 (21.3)	21 (32.3)	
Unknown	9 (5.1)	3 (4.6)	
Second line therapy			0.48
Axitinib	67 (37.6)	25 (38.5)	
Cabozantinib	85 (47.8)	33 (50.8)	
Sunitinib	8 (4.5)	1 (1.5)	
Pazopanib	18 (10.1)	5 (7.7)	
Sorafenib	0 (0)	1 (1.5)	

number(%)

*IMDC* International Metastatic Renal Cell Carcinoma Database Consortium, *IO* Immune-oncology, *TKI* tyrosine kinase inhibitor

The results of the primary treatments between the two categories are presented in Table 2. The best effects were 2.8% CR, 21.4% PR, 43.8% SD, and 28.1% PD in the NOG group, and 1.5% CR, 29.2% PR, 41.5% SD, and 26.2% PD in the OG group. No association was found between BMI and the occurrence of immune-related adverse events or grades ≥ 3 adverse events according to the Common Terminology Criteria for Adverse Events (P = 0.77 and P = 0.55, respectively).

**Table 2:** Treatment outcomes and treatment related AEs

Patients n = 243	BMI < 25	BMI ≥ 25	p
Number	178	65	
Best response			
CR	5 (2.8)	1 (1.5)	
PR	38 (21.4)	19 (29.2)	
SD	78 (43.8)	27 (41.5)	
PD	50 (28.1)	17 (26.2)	
Unknown	7 (3.9)	1 (1.5)	
irAE			
Yes	102 (57.3)	39 (60.0)	0.77
AEs grade 3 or more			
Yes	80 (44.9)	31 (47.7)	0.55

*CR* complete response, *PR* partial response, *SD* stable disease, *PD* progressive disease, *AE* adverse event, *irAE* immune-related adverse event

### Overweight patients

**Fig. 1:**
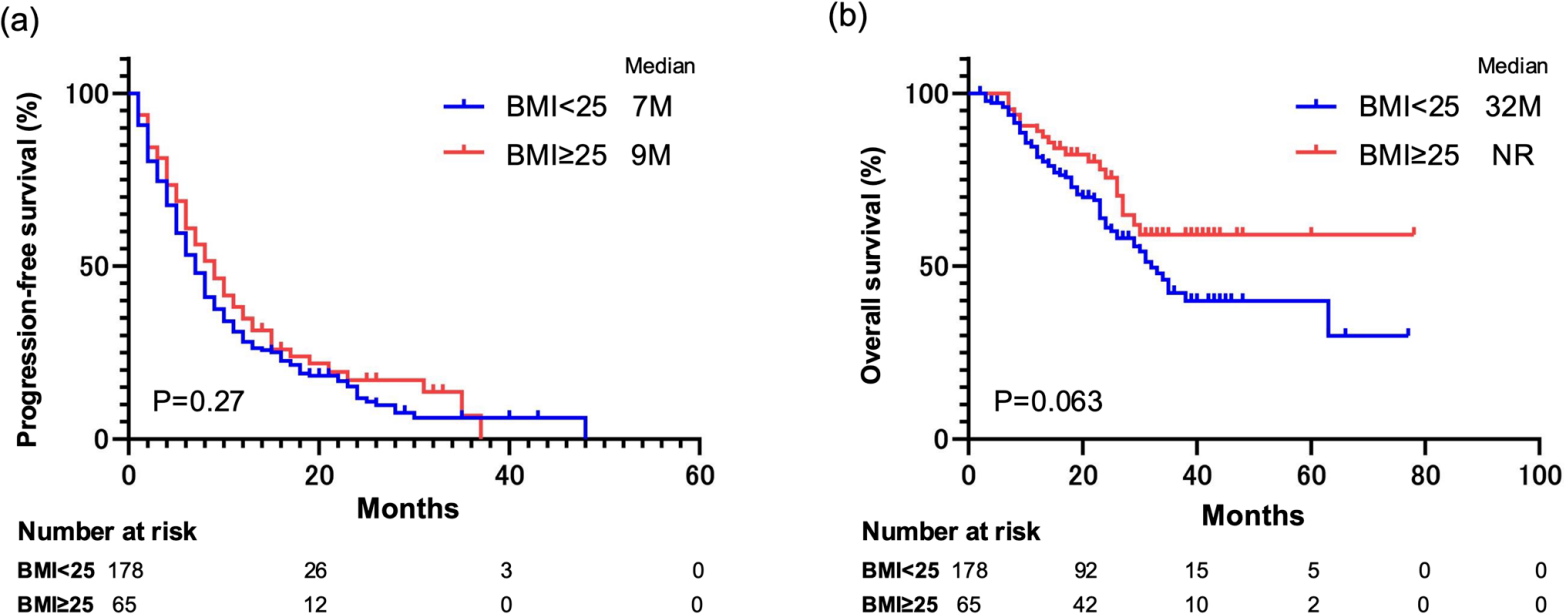
Survival curves illustrating the effect of body mass index (BMI) on combined immunotherapy outcomes. **a** Progression-free survival (PFS): Kaplan–Meier curves comparing PFS rates between patients with BMIs of < 25 vs those with BMIs of ≥ 25. **b** Overall survival (OS): Kaplan–Meier curves comparing OS rates between patients with BMIs of < 25 vs those with BMIs of ≥ 25

The PFS was 7 months in the NOG group and 9 months in the OG group, with almost identical curves between the two (P = 0.27; Fig. 1a). The OS was 32 months in the NOG group and not reached in the OG group. We observed a trend toward a better prognosis in the OG category, but this was not statistically significant (P = 0.063; Fig. 1b).

**Fig. 2:**
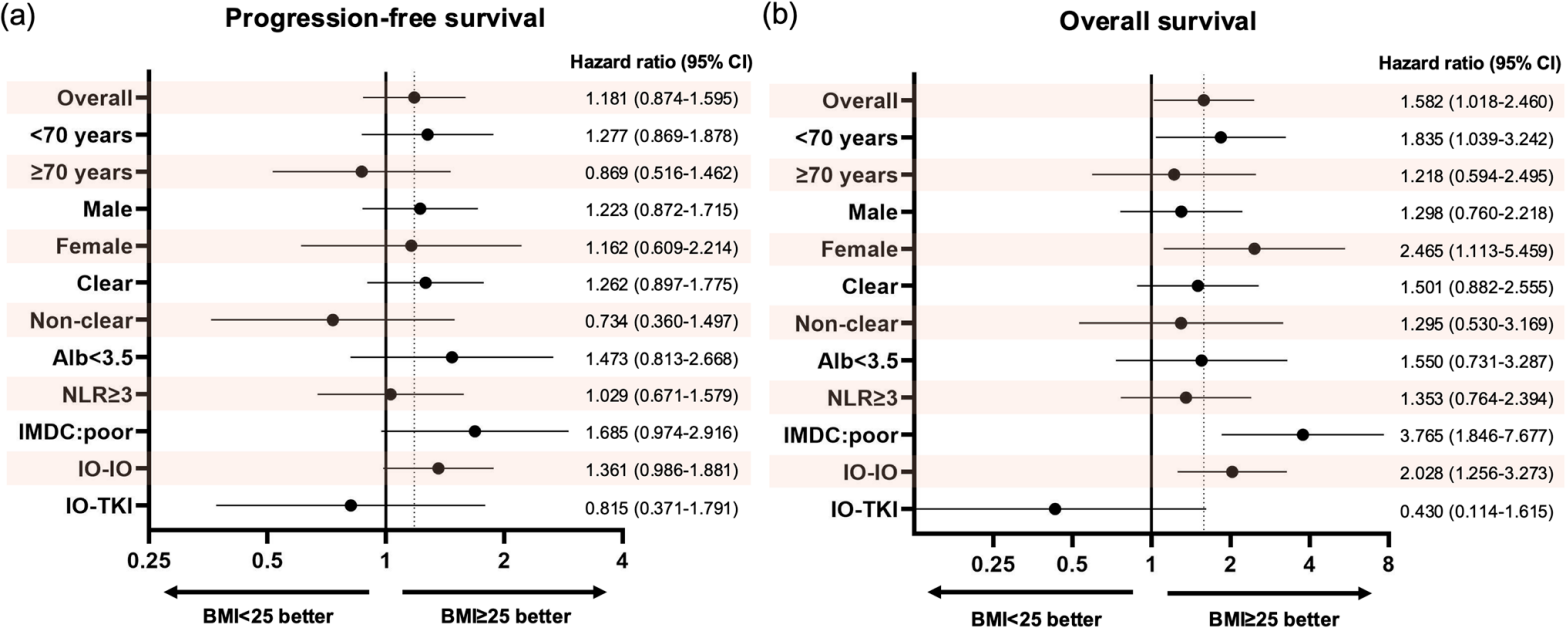
Forest plot comparing progression-free survival (PFS) and overall survival (OS) by body mass index (BMI). **a** Forest plot presenting hazard ratios (HRs) for PFS. **b** Forest plot presenting HRs for OS

Subgroup analyses were performed for PFS and OS (Fig. 2). We clarified the extent to which patient characteristics, such as age, sex, histological type, nutritional status, neutrophil/lymphocyte ratio, IMDC risk classification, and whether the regimen used was IO–IO or IO–TKI, affected PFS and OS in the NOG and OG categories. Regarding PFS, we found no significant differences according to whether a patient’s BMI value was < 25 kg/m^2^ or ≥ 25 kg/m^2^; however, an IO–IO regimen tended to be more effective in terms of OS within the subgroups of patients < 70 years of age, females, those with an IMDC risk of poor, and those with BMIs of < 25 kg/m^2^. In the subgroup with poor IMDC risk, the OG category had significantly better OS (Fig. S1), but this was not statistically significant in the < 70 years of age and female subgroups (Figs. S2, S3).

**Fig. 3:**
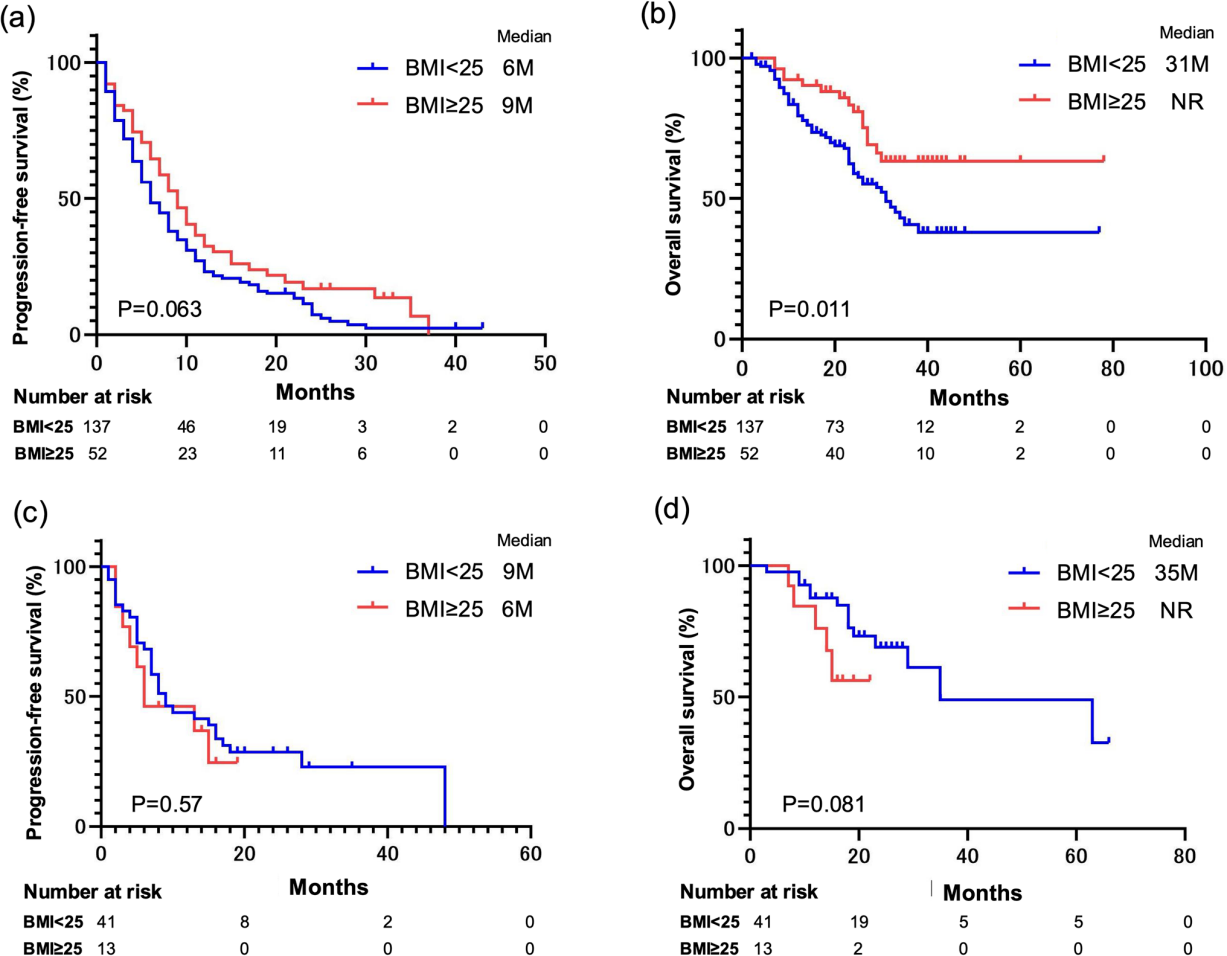
Survival analysis for each immune combination therapy regimen stratified by body mass index (BMI). **a** Kaplan–Meier curve for progression-free survival (PFS) in the BMI < 25 and BMI ≥ 25 categories of patients who received IO–IO therapy. **b** Kaplan–Meier curve for overall survival (OS) in the BMI < 25 and BMI ≥ 25 categories of patients who received IO–IO therapy. **c** Kaplan–Meier curve for PFS in the BMI < 25 and BMI ≥ 25 categories of patients who received IO–TKI therapy. **d** Kaplan–Meier curve for OS in the BMI < 25 and BMI ≥ 25 categories of patients who received IO–TKI therapy

Notably, opposing results were obtained when we considered the IO–IO vs IO–TKI treatment regimens. We compared the PFS and OS of patients who received IO–IO between the NOG and OG categories (Fig. 3a, b). Patient characteristics for those who received IO–IO and IO–TKI regimens are presented in Supplementary Tables 1 and 2, respectively. The OG category tended to have a better PFS, but the difference was not statistically significant (P = 0.063). In addition, the OG category had a significantly better OS (P = 0.011). We then compared the PFS and OS results of the patients who received IO–TKI between the NOG and OG categories (Fig. 3c, d), and found no difference in PFS (P = 0.57). The NOG category tended to have better OS, but this difference was not statistically significant (P = 0.081).

## Discussion

This study aimed to clarify the relationship between immune combination therapy and BMI in Japanese patients with metastatic RCC. Valuable insights were obtained regarding this intricate relationship.

We found that our patients in the OG group tended to have better prognoses; however, the difference was not statistically significant. This observation contradicts one in a previous report that the all-cause mortality rate rose by ~ 30% for every 5 kg/m^2^ increase in BMI [14], but is consistent with another regarding the “obesity paradox,” according to which the survival outcomes of IO–IO therapy for metastatic RCC are better in patients with higher BMIs [15]. Wang et al. demonstrated, in a series of murine experiments, that obesity causes immune aging, tumor progression, and T cell dysfunction mediated by the PD-1. This relationship was reported to be partially driven by leptin, which is associated with an increase in the efficacy of PD-1/PD-L1 blockade, emphasizing that obesity can represent a valuable biomarker for certain cancer immunotherapies [16]. Bader et al. reported the possibility of improving reactivity to PD-1 immunotherapy by showing that the metabolic and inflammatory signals associated with obesity induce PD-1 expression in macrophages [17]. Because the dataset used in this study was limited to clinical parameters and lucked molecular or immunological data, validating these findings was difficult. Although no basic mechanism related to obesity and CTLA4 immunotherapy has been reported, one clinical research report noted that the response rate was significantly higher in patients with BMIs of ≥ 25 kg/m^2^ who received ipilimumab monotherapy for metastatic malignant melanoma [18], suggesting that the underlying mechanism may be latent and clarified in future research.

There may be a relationship between irAE and the effect of immunotherapy. If immunotherapy is highly impactful in overweight patients, the irAE onset might be high. In a report that analyzed the relationship between irAE and BMI, based on this hypothesis, across a variety of cancers, patients with BMIs of ≥ 25 kg/m^2^ had significantly more irAEs (e.g., skin, endocrine, gastrointestinal, liver, and others) compared to their counterparts with normal weights [19]. However, in the present cohort, we found no difference in the onset of irAEs between the NOG and OG categories. This result may have been influenced by differences in the cohorts, as the aforementioned report [19] comprised only 14.2% patients with RCC, whereas the proportion of patients who fell into the overweight/obese category was 51%.

In the present study, a subgroup analysis was performed to examine how patient characteristics such as age, sex, histological type, nutritional status, neutrophil/lymphocyte ratio, IMDC risk classification, and treatment regimen used affected PFS and OS in the NOG and OG categories. Notably, the effect of BMI on prognosis clearly varied depending on the regimen. In the patients who were treated via IO–IO, the category with BMI values of ≥ 25 kg/m^2^ had significantly better OS. By contrast, those who received IO–TKI and had BMIs of < 25 kg/m^2^ tended to have better OS—although the difference was not statistically significant. These findings suggest that the relationship between BMI and the efficacy of IO–IO therapy may be influenced by the combination of drugs used during treatment. Sanchez et al. reported the non-inferiority of pazopanib to sunitinib in the COMPARZ trial cohort; their patients with obesity had longer survival times compared to their counterparts with normal weights, with the strongest association being observed in those who received sunitinib treatment [20]. In the same report, they analyzed differences in the transcriptomes of primary tumors between patients with obesity and those with normal weights. They found that the angiogenesis signaling pathway was significantly upregulated in the former group, which represents one reason why the “obesity paradox” has been established even for TKI treatment. In a report evaluating immune combination therapy for metastatic RCC by Santoni et al., OS was significantly better in the patient group with BMIs of ≥ 25 kg/m^2^, regardless of whether they were treated via IO–IO or IO–TKI [15]. Contrary to these reports, the results of this study demonstrated that the IO–TKI regimen had better OS in the patient category with BMI values of < 25 kg/m^2^—although this finding should be carefully re-examined in order to conclusively determine whether this represents a phenomenon that is unique to the Japanese population, or the result of a bias attributable to the study’s small sample size. Therefore, it would be beneficial to increase the number of cases and re-analyze the present ones in future related studies.

This study was subject to some key limitations worth noting. First, it was a retrospective study, indicating that it is inherently subject to various biases. Second, owing to the observation period, none of the included patients received the treatment regimens of cabozantinib + nivolumab or lenvatinib + pembrolizumab, which are currently popular for patients with metastatic RCC. Third, patients who achieved long-term success with only primary treatment, as well as those who faced challenges in continuing their treatments, were excluded. Therefore, this study cannot rule out the possibility that the therapeutic effects of secondary treatment may have influenced the outcomes. Finally, in addition to the main analysis of the entire population, subgroup analyses by treatment regimen (IO–IO group and IO–TKI group) were also conducted. However, due to the limited number of cases in each subgroup, sufficiently robust analyses could not be performed. Validation using a larger cohort is warranted in future studies.

The results of this study will hopefully contribute to a better understanding of the factors affecting the efficacy of immune combination therapy in patients with RCC. This knowledge may help to optimize treatment strategies for this deadly malignancy, by considering each patient’s BMI and the specific treatment regimen being proposed. However, further research is warranted to elucidate the mechanisms underlying the observed “obesity paradox,” as well as to confirm our findings in larger patient cohorts.

## Supplementary Information

Below is the link to the electronic supplementary material.

Supplementary file1 Fig. S1: Subgroup analysis of immune combination therapy by BMI in the IMDC risk: poor patient group. (a) Progression-free survival (PFS): Kaplan–Meier curves comparing PFS between patients with BMI < 25 and those with BMI ≥ 25. (b) Overall survival (OS): Kaplan–Meier curves comparing OS rates between patients with BMIs of < 25 vs those with BMIs of ≥ 25 (TIFF 35159 KB)

Supplementary file2 Fig. S2: Subgroup analysis of immune combination therapy by BMI in the patient group aged < 70 years. (a) Progression-free survival (PFS): Kaplan–Meier curves comparing PFS rates between patients with BMIs of < 25 vs those with BMIs of ≥ 25. (b) Overall survival (OS): Kaplan–Meier curves comparing OS rates between patients with BMIs of < 25 vs those with BMIs of ≥ 25 (TIFF 35159 KB)

Supplementary file3 Fig. S3: Subgroup analysis of immune combination therapy by BMI in the female patient group. (a) Progression-free survival (PFS): Kaplan–Meier curves comparing PFS rates between patients with BMIs of < 25 vs those with BMIs of ≥ 25. (b) Overall survival (OS): Kaplan–Meier curves comparing OS rates between patients with BMIs of < 25 vs those with BMIs of ≥ 25 (TIFF 35159 KB)

Supplementary file4 (XLSX 15 KB)

## Data Availability

The data analyzed in this research are not publicly available; however, they can be obtained from the corresponding author upon reasonable request.
