# Supplementary figures and images for: Impact of body mass index on the efficacy of immune combination therapy in metastatic renal cell carcinoma: a multicenter study in Japan
Source: Int J Clin Oncol. 2025 Aug 20;30(10):2079–86. doi: 10.1007/s10147-025-02823-0 (PMC12474707; doi:10.1007/s10147-025-02823-0)

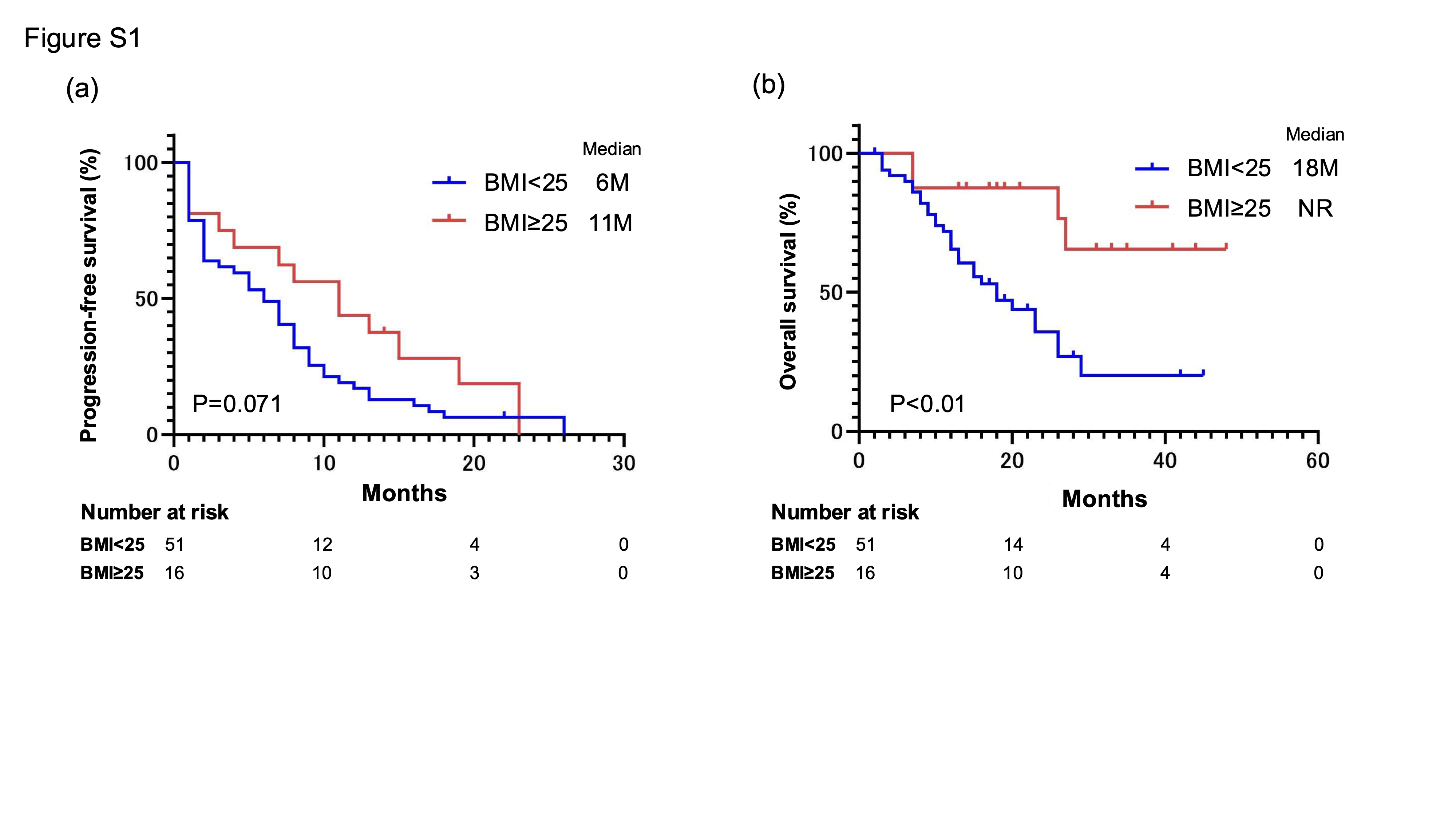

Supplement: Supplementary file 1 — Supplementary file1 Fig. S1: Subgroup analysis of immune combination therapy by BMI in the IMDC risk: poor patient group. (a) Progression-free survival (PFS): Kaplan–Meier curves comparing PFS between patients with BMI < 25 and those with BMI ≥ 25. (b) Overall survival (OS): Kaplan–Meier curves comparing OS rates between patients with BMIs of < 25 vs those with BMIs of ≥ 25 (TIFF 35159 KB) [file 10147_2025_2823_MOESM1_ESM.tiff]

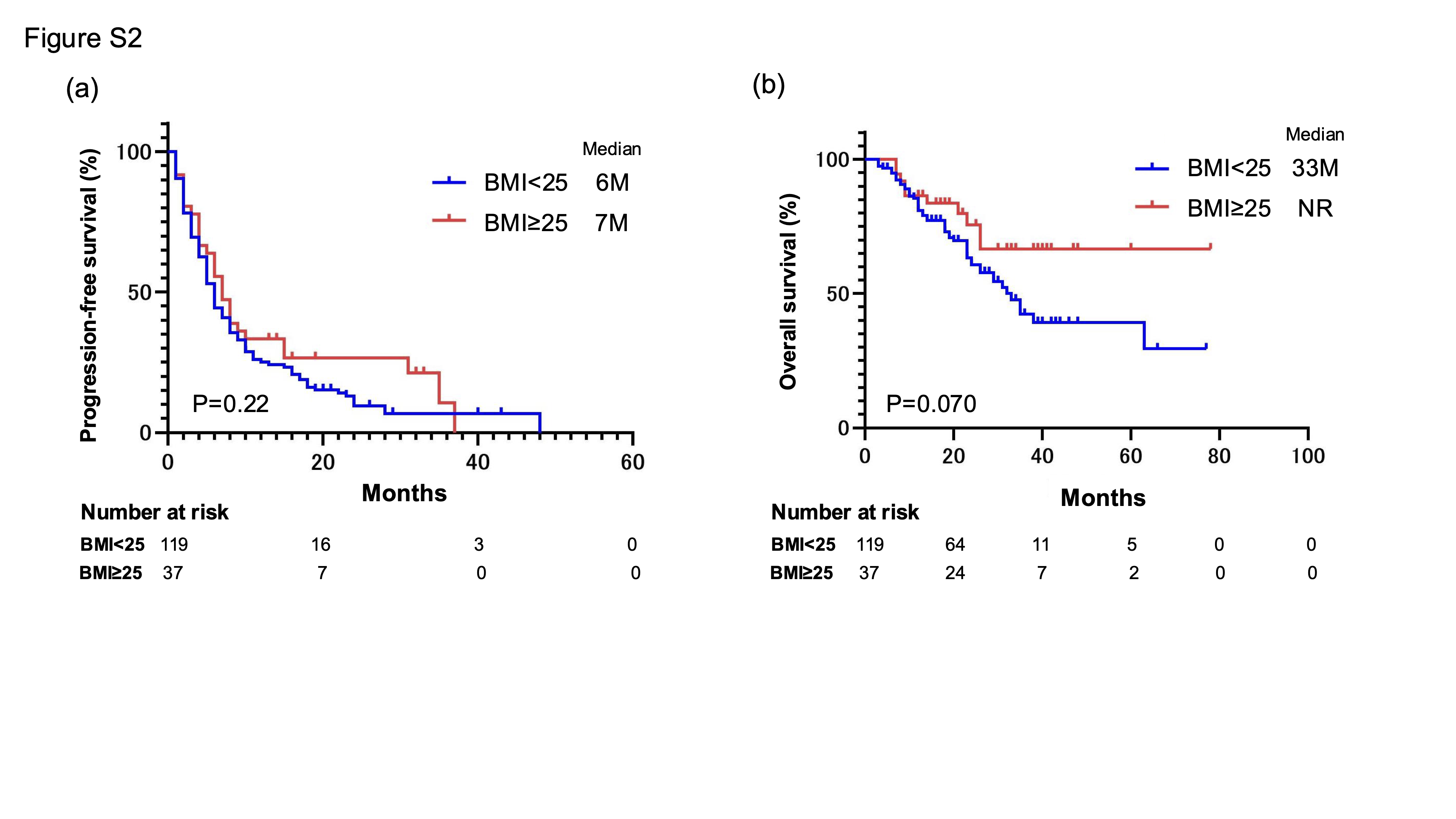

Supplement: Supplementary file 2 — Supplementary file2 Fig. S2: Subgroup analysis of immune combination therapy by BMI in the patient group aged < 70 years. (a) Progression-free survival (PFS): Kaplan–Meier curves comparing PFS rates between patients with BMIs of < 25 vs those with BMIs of ≥ 25. (b) Overall survival (OS): Kaplan–Meier curves comparing OS rates between patients with BMIs of < 25 vs those with BMIs of ≥ 25 (TIFF 35159 KB) [file 10147_2025_2823_MOESM2_ESM.tiff]

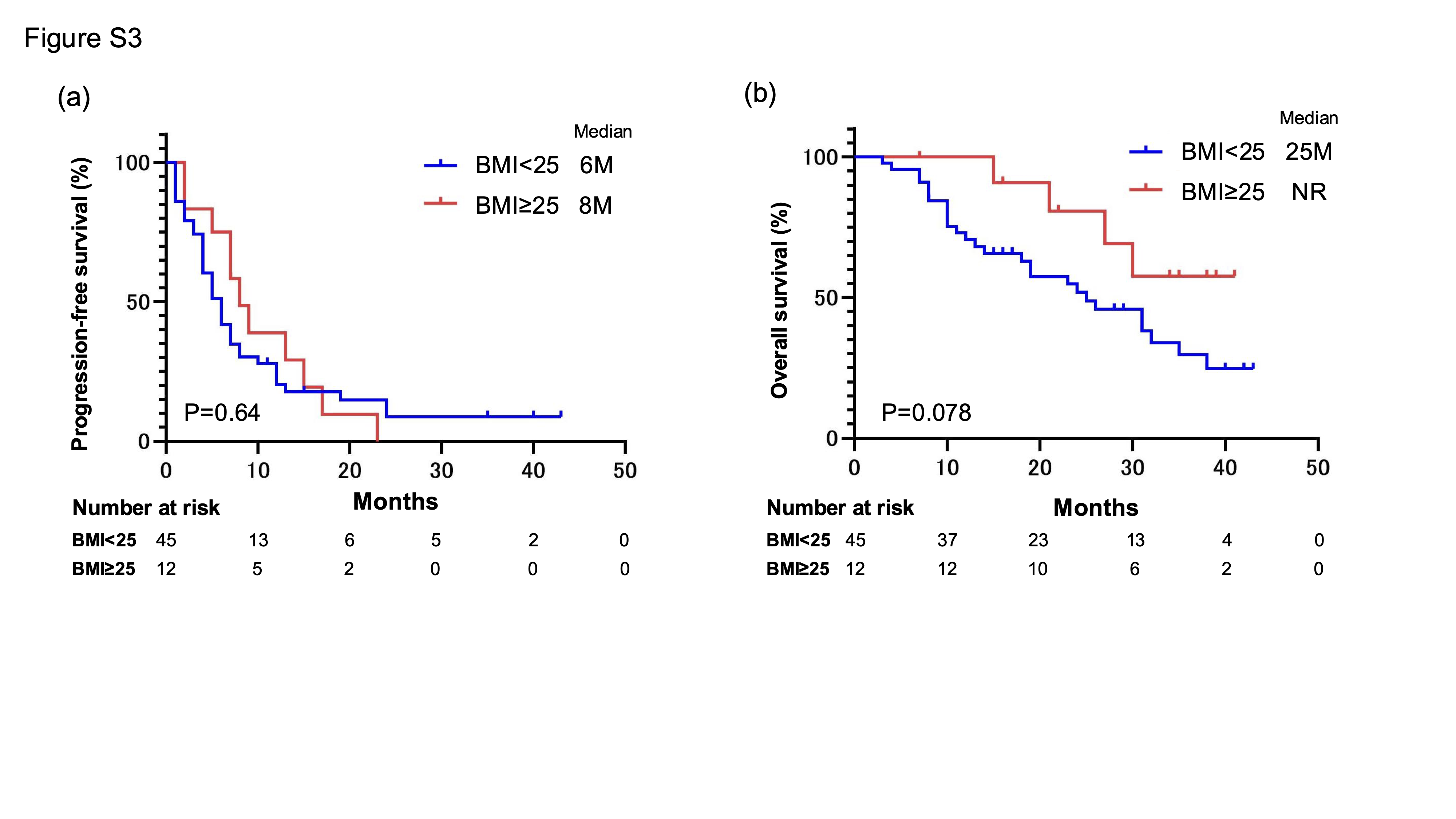

Supplement: Supplementary file 3 — Supplementary file3 Fig. S3: Subgroup analysis of immune combination therapy by BMI in the female patient group. (a) Progression-free survival (PFS): Kaplan–Meier curves comparing PFS rates between patients with BMIs of < 25 vs those with BMIs of ≥ 25. (b) Overall survival (OS): Kaplan–Meier curves comparing OS rates between patients with BMIs of < 25 vs those with BMIs of ≥ 25 (TIFF 35159 KB) [file 10147_2025_2823_MOESM3_ESM.tiff]
